# Mechanistic basis and clinical relevance of the role of transforming growth factor-β in cancer

**DOI:** 10.7497/j.issn.2095-3941.2015.0015

**Published:** 2015-12

**Authors:** Run-Long Lin, Lu-Jun Zhao

**Affiliations:** Department of Radiation Oncology, Tianjin Medical University Cancer Institute and Hospital, National Clinical Research Center for Cancer, Key Laboratory of Cancer Prevention and Therapy, Tianjin 300060, China

**Keywords:** Transforming growth factor-β (TGF-β), neoplasms, prognosis, therapeutics

## Abstract

Transforming growth factor-β (TGF-β) is a key factor in cancer development and progression. TGF-β can suppress tumorigenesis by inhibiting cell cycle progression and stimulating apoptosis in early stages of cancer progression. However, TGF-β can modulate cancer-related processes, such as cell invasion, distant metastasis, and microenvironment modification that may be used by cancer cells to their advantage in late stages. Corresponding mechanisms include angiogenesis promotion, anti-tumor immunity suppression, and epithelial-to-mesenchymal transition (EMT) induction. The correlation between TGF-β expression and cancer prognosis has also been extensively investigated. Results suggest that TGF-β pathway can be targeted to treat cancer; as such, the feasibility of this treatment is investigated in clinical trials.

## Introduction

According to GLOBOCAN estimates, approximately 14.1 million new cancer cases and 8.2 million deaths occurred in 2012 worldwide^1^. Transforming growth factor-β (TGF-β) plays an important role in epithelial and neural tissue development, immune system regulation, and wound repair^2^. TGF-β also participates in cancer development and progression.

TGF-β can suppress tumorigenesis by inhibiting cell cycle progression and inducing apoptosis. Specific mechanisms that mediate the cytostatic effect of TGF-β have been mainly investigated in epithelial cell types. TGF-β inhibits the progression of G_1_ phase via two events shared by skin, lung, and mammary epithelial cells: (I) mobilization of cyclin-dependent kinase (CDK) inhibitors; and (II) suppression of proto-oncogene c-MYC and ID proteins. Furthermore, the ability of TGF-β to induce apoptosis varies greatly depending on cell type. However, our knowledge of this area remains limited.

Malignant cells can evade suppressive effects of TGF-β and use TGF-β regulatory functions to their advantage. Thus, these cells acquire invasive and metastatic capabilities via angiopoiesis, immune surveillance suppression, epithelial-to-mesenchymal transition (EMT) promotion, and extracellular matrix (ECM) degradation.

**Figure 1:**
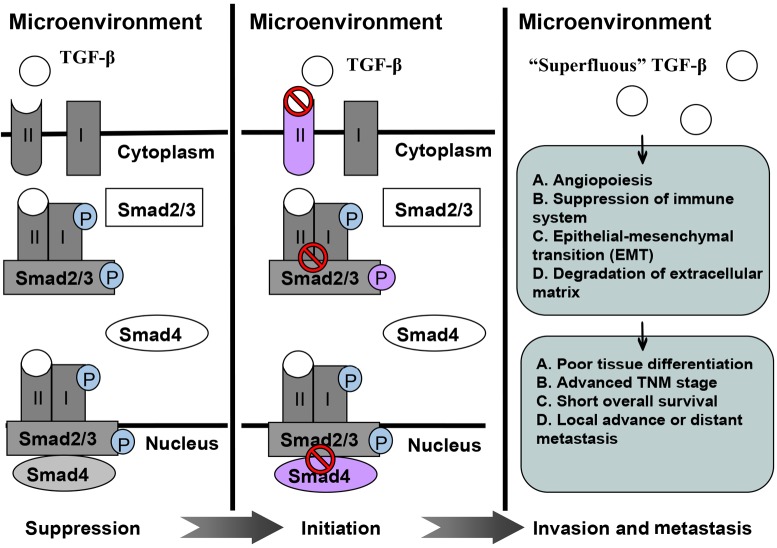
A schematic illustrating role of TGF-β in tumor suppression, initiation, invasion, and metastasis.

TGF-β expression is correlated with poor tissue differentiation, advanced TNM stages, short overall survival, and locally advanced or distant metastasis in various malignancies. However, controversial findings should be considered. TGF-β inhibition can elicit anti-tumor effect, especially when this mechanism is combined with radiotherapy; TGF-β levels increase. Preliminary clinical trials have been performed to evaluate the feasibility of TGF-β inhibitors as anti-tumor modalities. This review summarizes recent advances in this area and discusses potentially relevant mechanisms. **Figure 1** shows a schematic of the role of TGF-β in malignancy development.

## TGF-β pathway

The TGF-β superfamily includes TGF-β ligands (TGF-β1, -2, and -3), bone morphogenic proteins (BMPs), and activins/inhibins. TGF-β factors are synthesized as dimeric prohormones and secreted into ECM. Canonical TGF-β/Smad signaling cascade is initiated when a TGF-β/BMP ligand binds to type II serine/threonine kinase receptors; type II serine/threonine kinase receptors in turn recruit and phosphorylate type I receptors^3^. Phosphorylated type I receptors then propagate a signal by phosphorylating RSmad, which forms a complex with Smad4. TGF-β ligands transmit the signal through Smad2 and Smad3, whereas BMPs employ Smad1, Smad5, and Smad8^4^^,^^5^. Activated Smad complexes are transported into the nucleus, where these complexes, together with co-activators or co-repressors, regulate target gene transcription^2^. In non-canonical TGF-β signaling, ligand-bound TGF-β receptors activate other signaling pathways, such as p38 and Jun N-terminal kinase (JNK) mitogen-activated protein kinase (MAPK) pathways, phosphoinositide 3-kinase-Akt-mTOR pathway, small GTPase RhoA and Rac/Cdc42 pathways, and Ras-Erk pathway; the activation of these pathways likely enhances tumor growth after canonical TGF-β-Smad signaling is disrupted^6^^,^^7^.

## Tumor-suppressive effect of TGF-β

### Cytostatic mechanisms

Cell division cycle proceeds by the action of CDKs. TGF-β action inhibits CDKs, which stimulate the G_1_ phase of the cell cycle. In epithelial cells, TGF-β induces the expression of p15^Ink4b^, which inhibits cyclinD-CDK4/6 complexes; TGF-β also causes the expression of p21^Cip1^, which inhibits cyclinE/A-CDK2 complexes^8^^,^^9^. In addition, TGF-β/Smad signaling stimulates p15^Ink4b^ expression, thereby promoting the release of inactive p27^Kip1^ from cyclin D-CDK4 complexes to allow the p27^Kip1^-dependent inhibition of cyclin E/A-CDK2 complexes^10^. TGF-β also induces p16^ink4a^ and p19^ARF^ expression; this induction contributes to growth arrest and senescence response^11^. Furthermore, TGF-β reduces CDK activity; as a result, cyclin/CDK complex-induced phosphorylation of the tumor suppressor Rb protein is prevented. Thus, hypophosphorylated Rb can bind to transcription factors in the E2F family and impede the ability of these factors to promote G_1_/S phase cell cycle progression^12^.

Another important event in the TGF-β anti-proliferative program is the inhibition of c-MYC expression. c-MYC can bind to an initiator element of p15^Ink4b^ promoter; as a consequence, p15^Ink4b^ expression is inhibited^13^. mRNA and protein levels of c-MYC rapidly decrease in response to TGF-β because the corresponding mRNA and protein are short- lived; thus, c-MYC-mediated p15^Ink4b^ repression is relieved^14^. Comparing the genome-wide profile of TGF-β gene responses in non-tumorigenic and tumor-derived human mammary cells, Chen *et al.*^15^ noted that the loss of TGF-β growth inhibitory effect in tumor-derived human mammary cells occurs with a selective loss of the corresponding c-MYC down-regulation.

TGF-β can inhibit ID1 and ID2 expression, in addition to c-MYC down-regulation. ID proteins function as negative regulators implicated in cell differentiation. In ID1 down-regulation, TGF-β stimulation rapidly induces the expression of activating transcription factor-3 (ATF3) in HaCaT skin keratinocytes, HPL1 lung epithelial cells, and MCF-10A mammary epithelial cells via a Smad3-containing transcriptional complex. ATF3 then coordinates with Smad3 and Smad4 to target ID1 and repress transcription^16^. A link between c-MYC and ID2 expression has been established by showing that the binding of c-MYC to E-box motifs in ID2 promoter supports ID2 expression. Thus, TGF-β-induced c-MYC down-regulation may also inhibit ID2 in specific cell types^17^.

### Pro-apoptotic mechanisms

p38 MAPK activator DAXX is considered as a mediator of TGF-β apoptotic signals because this molecule physically interacts with TGF-β receptor and requires TGF-β-induced apoptosis. The carboxyl terminal of DAXX functions as a dominant negative inhibitor of TGF-β-induced apoptosis in B cell lymphomas; antisense oligonucleotides against DAXX also inhibit TGF-β-induced apoptosis in mouse hepatocytes^18^. The expression of the death-associated protein kinase (DAP-kinase) is increased during TGF-β-induced apoptosis in hepatoma cells. Overexpression of DAP-kinase triggers apoptosis in the absence of TGF-β, whereas inhibition of DAP-kinase activity protects cells from TGF-β-induced apoptosis. And this process relies on Smad function and sensitizes cells to undergo TGF-β/Smad apoptosis^19^. Apoptosis-related protein in the TGF-β signaling (ARTS) pathway is an unusual mitochondrial septin-like protein that functions as a tumor suppressor. In an embryonic kidney cell line, ARTS potentiates apoptosis in cells normally resistant to TGF-β-induced cell death; by contrast, reduced ARTS expression impairs TGF-β-induced apoptosis^20^.

## Tumorigenic effects of TGF-β on premalignant cells

### TGF-β receptor mutation

The loss of canonical TGF-β/Smad signaling pathway initiates tumors; this phenomenon occurs because some tumor cells may escape the inhibitory effects of the canonical pathway through mutations that can provide a growth advantage over benign tumors^21^. TGF-β receptors (TGF-β R-I and TGF-β R-II) are required for the proper transduction of TGF-β signaling pathway. Tumor cells may reduce the expression or function of either TGF-β R-I or TGF-β R-II to escape growth-inhibiting effects of TGF-β canonical pathway. This TGF-β receptor-induced loss of sensitivity to TGF-β canonical pathway can cause compensatory TGF-β overexpression; as a result, aggressive effects are elicited^22^. The loss of TGF-β R-II on myeloma cell surface can also be attributed to gene silencing through hypermethylation; this loss can promote myeloma cell resistance to anti-cancer effects^23^. TGF-β R-II mutations have also been described in association with microsatellite instable (MSI^+^) carcinomas. In MSI^+^ cells, DNA base mismatch repair is compromised; thus, errors in a 10 bp poly-adenine repeat segment from the coding region [Poly(A) 10 tract] of TGF-β R-II are frequently observed. These mutations often lead to frame-shift missense mutations or early termination that prevents proper translation of a functional TGF-β R-II protein^24^.

### Disruption of downstream canonical signaling pathway

In addition to the crucial role of the canonical signaling pathway in RSmad (Smad2/Smad3) phosphorylation, the role of the activation of the non-canonical pathway by TGF-β receptors likely create phospho-specific Smad2/Smad3. Non-canonical phosphorylation of the RSmads (Smad2/Smad3) via non-canonical pathways, such as MAPK pathway, can inhibit canonical phosphorylation induced by TGF-β receptor complex and disrupt anti-cancer effects of the canonical pathway^25^. Smad2/Smad3 phosphorylation via the MAPK pathway is possibly implicated in the transformation of oncogenic pSmad2/3L into tumor suppressor pSmad2/3C^26^. Frequent Smad4 gene mutation is associated with poorer prognosis of colorectal adenocarcinoma^27^, pancreatic cancer^28^, and papillary thyroid carcinoma progression^29^. With canonical TGF-β/Smad signaling, Smad7 uses a negative feedback mechanism to disrupt Smad2/3 phosphorylation through the competitive inhibition of TGF-β receptor complex; Smad7 also disrupts Smad2/3/4 complex formation and nuclear translocation by recruiting ubiquitin ligases that induce proteasomal degradation^30^.

## Promotive effect of TGF-β on tumor invasion and metastasis

Genetic mutations and downstream alterations in TGF-β/Smad signaling components often inactivate growth inhibitory activities of TGF-β; thus, TGF-β can contribute to cancer progression^31^^,^^32^. Related mechanisms include angiogenesis promotion, anti-tumor immunity suppression, and EMT induction.

### Angiopoiesis

New blood vessel formation in tumor tissues (tumor angiogenesis) is necessary to promote tumor cell growth and metastasis. The role of TGF-β in the angiogenesis of cancer cells is highly complex; this function involves the interaction of vascular endothelial growth factor (VEGF) and endothelin.

Soufla *et al.*^33^ demonstrated that VEGF and TGF-β are involved in endometrial carcinogenesis through transcription activation and down-regulation, respectively; Soufla *et al.*^33^ also suggested the potential use of VEGF and TGF-β as molecular indicators of disease progression. Tumor-derived VEGF-A triggers enhanced tumor cell proliferation possibly through the paracrine inhibition of TGF-β signaling within a tumor^34^.

Endoglin is a transmembrane glycoprotein involved in several processes and disorders associated with human circulatory system^35^. Endoglin acts as a type I transmembrane protein that functions as a co-receptor of TGF-β to modulate signaling by binding to TGF-β receptors and impairing downstream signaling activity^36^. TGF-β expression is also associated with tumor progression, as in the case of endoglin. TGF-β predicts poorer survival in endoglin-enriched tumors; this phenomenon indicates that TGF-β enhances disease progression in later stages of angiogenesis^36^^,^^37^. Previous studies with experimental models showed that highly expressed endoglin antagonizes the inhibitory effects of TGF-β and contributes to proliferation, migration, and capillary formation of endothelial cells, which are the three key events in angiogenesis. TGF-β also plays an important role in angiogenesis by promoting endothelial cell proliferation and migration at low concentrations and by causing vessel maturation at high concentrations^38^.

### Immune system suppression

TGF-β-induced immunosuppression occurs when tumor cells escape attack by the immune system through various mechanisms, including interaction with CD4^+^CD25^+^Foxp3^+^ regulatory T cells (Treg cells), tumor-associated macrophages, tumor-associated neutrophils (TAN), and T helper 17 (TH17).

CD8^+^CTLs (cytotoxic T lymphocytes) are necessary to control tumor progression. Treg cells are a specialized T cell subpopulation, which suppresses immune system activation^39^. In various tumor types, natural and adaptive Treg cell concentrations are increased in tumor sites and contribute to tumor-induced immunosuppression by suppressing CTL proliferation and function^40^. *In vitro* experiments have revealed that the TGF-β secreted by a renal carcinoma cell line and a prostate cancer cell line can induce the transformation of CD4^+^CD25^−^ T cells in mouse spleen into Treg cells^41^. The extent of Treg cell infiltration is higher in high-TGF-β-expression group than in low-TGF-β-expression group; this result indicates that TGF-β expression in tumor tissues can increase Treg cell infiltration in a local tumor; thus, tumor cells evade immune responses^42^. Lu *et al.*^43^ showed that gastric cancer-induced infiltration of Treg cells predicts the poor prognosis of patients with gastric adenocarcinoma; some of these Treg cells are converted by tumor-produced TGF-β.

Macrophages are also important immune cells in peripheral blood. Macrophages are necessary to prevent metastasis of cancer cells. Classically activated M1 macrophages can phagocytose tumor cells. Therefore, these macrophages are involved in immune function against infection and tumor cell invasion. M1 macrophages also play a critical role in cellular immunity against cancer. Alternatively activated M2 macrophages perform a distinct function from M1 macrophages. M2 macrophages can facilitate tumor cell proliferation, angiogenesis, and tissue remodeling. These effects are mainly achieved through TGF-β secretion^44^. Other mechanisms, such as TAN and Th17 pathways, associated with the anti-tumor immune effect of the TGF-β pathway have been reported^45^^,^^46^.

### TGF-β pathway and EMT

Tumor invasion and metastasis are initiated by decreased cell-to-cell adhesion, increased motility, and invasive properties that allow carcinoma cells to detach from primary tumor and invade surrounding tissues through collective or individual cell migration. TGF-β functions as a potent stimulator of cancer progression by inducing EMT; in this process, epithelial cells acquire a mesenchymal phenotype and exhibit enhanced motility and invasion^47^. Cells undergoing EMT down-regulate the expression of E-cadherin epithelial marker and increase the expression of N-cadherin, a mesenchymal marker^48^. Cells can respond to TGF-β through growth inhibition and EMT. Pino *et al*.^49^ reported that TGF-β induces EMT in colon cancer cell lines with a wild-type TGF-β R-II. However, no changes in cell morphological characteristics, differentiation marker expression, motility, and invasion have been observed in cells with homozygous TGF-β R-II mutations. This finding reveals that growth inhibition and EMT may share canonical TGF-β/Smad pathway as a common signaling pathway.

TGF-β levels are positively associated with tumor resistance to radiotherapy or chemotherapy; this positive association may attribute to treatment-initiated EMT of tumor cells. Zhao *et al.*^50^ observed that increased TGF-β levels during radiation therapy are strongly correlated with poor prognosis among patients with non-small cell lung cancer. In addition, poor prognosis of glioblastoma (GBM) routinely treated with ionizing radiation has been attributed to the relative radioresistance of glioma-initiating cells (GICs). GICs are sensitive to treatment, but response is mediated by undefined factors in a microenvironment. GIC resistance to radiation, which is mediated by a tumor microenvironment, can be abolished by inhibiting TGF-β/Smad signaling pathway^51^. Tas *et al*.^52^ showed that patients with chemotherapy-unresponsive epithelial ovarian cancer present higher serum TGF-β levels than responsive patients (*P*=0.02). These studies support the current hypothesis that a subtle relationship exists among TGF-β, EMT phenotype, and therapy resistance. TGF-β may be a new molecular subtype that can cause resistance to therapy.

### ECM degradation

Tumor ECM degradation is a critical step in tumor invasion and metastasis. TGF-β plays an important role in ECM degradation. ECM is mainly degraded by proteolytic enzymes, such as matrix metalloproteinases (MMPs). MMPs are a group of proteolytic enzymes that can degrade tumor ECM and are up-regulated in several tumor tissues. Yang *et al.*^53^ found that TGF-β expression levels exhibit a significantly positive correlation with MMP2 expression in renal clear cell carcinoma. A similar finding has been observed in melanoma^54^. TGF-β is also significantly correlated with MMP9 expression; MMP9 can facilitate tumor cell infiltration in lymphatic or blood systems by degrading basement membrane components^55^. This effect is another mechanism in the tumor-promoting effect of TGF-β.

## Clinical significance of TGF-β

TGF-β plays an important role in cancer development and progression. TGF-β expression may predict the prognosis of patients with malignancy. Studies have investigated the prognostic role of TGF-β protein/mRNA expression in cancer. Some of the related studies are summarized in **Table 1**. These studies have indicated that a high TGF-β expression may predict poor prognosis, including poor tissue differentiation, advanced TNM stage, short overall survival, and locally advanced or distant metastasis. However, other studies have revealed controversial findings. Discrepancies may be attributed to several factors, such as differentiation status of analyzed tumors and disease stage. TGF-β pathway regulation occurring post-transcription may differ among samples. Discrepancies may also be caused by different methods used to detect TGF-β expression.

**Table 1:** Relevance analysis between TGF-β expression and prognosis

Author (Year)	Tumor type	Patient number	Sample type	Detection method	Relevance with higher expression
Robson *et al.*^56^ (1996)	Colorectal adenocarcinoma	72	Biopsy specimen/protein	IHC	Shorter overall survival
Saito *et al*.^57^ (1999)	Gastric carcinoma	101	Biopsy specimen/protein	IHC	Poorer pathological pattern/higher expression of VEGF/higher MVD
Boldrini *et al.*^58^ (2000)	Non-small cell lung carcinoma	61	Biopsy specimen/mRNA	PCR	Longer overall survival
Hasegawa *et al.*^59^ (2001)	Non-small cell lung carcinoma	53	Biopsy specimen/protein	ELISA	More advanced T or N stage/higher MVD/shorter overall survival
Hashimoto *et al.*^60^ (2001)	Invasive ductal carcinoma of the pancreas	62	Biopsy specimen/protein	IHC	Shorter overall survival
Logullo *et al.*^61^ (2003)	Head and neck squamous cell carcinoma	140	Biopsy specimen/protein	IHC	Non-statistically significant survival rates in 5 years
Fukuchi *et al.*^62^ (2004)	Esophageal Cancer	57	Blood/protein	ELISA	More advanced N stage/shorter overall survival
Okumoto *et al.*^63^ (2004)	Hepatocellular carcinoma	70	Blood/protein	ELISA	Lower natural killer and lymphokine-activated killer cells
von Rahden *et al.*^64^ (2006)	Esophageal cancer	123	Biopsy specimen/mRNA	PCR	Poorer pathological pattern/more advanced T or N stage
Zhao *et al*.^50^ (2010)	Non-small cell lung carcinoma	65	Blood/protein	ELISA	Shorter overall survival and progression-free survival
Reis *et al*.^65^ (2011)	Prostate cancer	100	Biopsy specimen/mRNA	PCR	Gleason score
Valkov *et al.*^66^ (2011)	Soft tissue sarcoma	249	Biopsy specimen/protein	IHC	Shorter disease-specific survival
Dave *et al.*^67^ (2012)	Breast cancer	117	Blood/protein	ELISA	More advanced clinical stage/shorter overall survival
Fan *et al*.^68^ (2012)	Cervical squamous cell carcinoma	91	Biopsy specimen/protein	IHC	Deeper infiltration/worse pathological pattern/more advanced N stage
Divella *et al*.^69^ (2013)	Breast cancer	61	Blood/protein	ELISA	Higher rate of distant metastasis
Javle *et al.*^70^ (2014)	Pancreatic ductal adenocarcinoma	644	Blood/protein	96-well multi-array human TGF-β assay kit	Shorter survival in patients with locally advance or distant metastasis

## Opportunities and challenges in therapeutically targeting TGF-β

Targeting TGF-β may elicit a significant anti-tumor effect because TGF-β is implicated in cancer development and progression. TGF-β inhibitors have been preclinically evaluated; some of these inhibitors are in early stage clinical studies. Preliminary studies are summarized in **Table 2**. TGF-β inhibition among cancer patients has also been evaluated through clinical trials by using an antibody (GC1008) or an oligonucleotide (AP12009). These trials suggest that TGF-β inhibition exhibits promising efficacy and safety. However, large clinical trials should be conducted to clarify the feasibility and safety of treatments. Other challenges related to this approach include targeting a tumor microenvironment by using TGF-β inhibitors without affecting TGF-β function in hosts to maintain systemic homeostatic processes.

**Table 2:** Summary of TGF-β inhibitors as novel therapeutic targets

Drug	Mechanism of action	Development stage	Malignancy type	Referenced summary of results
AP12009	Antisense oligodeoxynucleotide specific for the mRNA of human TGF-β2	Phase I/II	Glioblastoma/anaplastic astrocytoma	Superior efficacy and safety for AP12009 over chemotherapy and positive risk-benefit assessment^71^
CAT192 (lerdelimumab)	Monoclonal antibody to TGF-β1	Preclinical	Human lung epithelial cells (A549)	An approximate median inhibitory concentration (IC_50_) value of 3 mg/mL^72^
GC1008	Monoclonal antibody to TGF-β1	Phase I	Advanced renal cell carcinoma or malignant melanoma	Safe and well tolerated^73^
AP11014	Antisense oligodeoxynucleotide specific for the mRNA of human TGF-β1	Preclinical	Lung, colon, and prostate cancer cell lines	Decreased TGF-β secretion^73^
ID11	Monoclonal antibody to TGF-β	Preclinical	Breast cancer cell lines	Suppressed breast cancer metastases to lungs^74^

## Conclusion

TGF-β functions as a trigger of a canonical suppression pathway, inducer of tumor angiogenesis, tumor-derived immunosuppressor, promoter of carcinoma invasion and metastasis, and influencing factor of chemotherapy and radiotherapy; thus, the dual role of TGF-β has been extensively investigated. TGF-β levels determined during diagnosis and treatment may also be a reliable marker; this method may be used to predict the prognosis of patients with cancer. High TGF-β1 levels are also associated with poor tissue differentiation, advanced TNM stage, and decreased overall survival. TGF-β inhibitors, especially those used in late cancer stage, may elicit anti-tumor effects via novel mechanisms, such as tumor angiogenesis suppression, immune system promotion, and EMT reversal. However, the optimal timing of TGF-β blockade and the ideal combination of this approach with other therapies, such as radiotherapy and chemotherapy, remain unknown. These questions are currently addressed in ongoing preclinical studies and will be resolved in future clinical trials.
